# Bi-directional differentiation of single bronchioalveolar stem cells during lung repair

**DOI:** 10.1038/s41421-019-0132-8

**Published:** 2020-01-07

**Authors:** Kuo Liu, Muxue Tang, Qiaozhen Liu, Ximeng Han, Hengwei Jin, Huan Zhu, Yan Li, Lingjuan He, Hongbin Ji, Bin Zhou

**Affiliations:** 1 State Key Laboratory of Cell Biology, CAS Center for Excellence in Molecular Cell Science, Institute of Biochemistry and Cell Biology, Shanghai Institutes for Biological Sciences, University of Chinese Academy of Sciences, Chinese Academic of Sciences, Shanghai, 200031 China; 2 School of Life Science and Technology, ShanghaiTech University, Shanghai, 201210 China

**Keywords:** Multipotent stem cells, Pluripotency

Dear Editor,

The lung is a multi-functional organ that executes gas exchange and innate defense functions. The epithelium of respiratory tree consists of four regions from proximal to distal: trachea, bronchi, bronchiole, and alveolar regions. Multiple stem or progenitor cells are required for maintaining lung functions during normal condition and repair. The maintenance and repair of epithelium at different regions mainly depend on its resident stem or progenitor cells^1–3^.

Recently, a new type of multipotent stem cells termed bronchioalveolar stem cells (BASCs) has been identified which located at the bronchioalveolar-duct junctions (BADJs)^4,5^. BASCs coexpress club cell maker secretoglobin 1a1 (Scgb1a1 or CC10) and AT2 cell maker surfactant protein C (Sftpc or SPC)^4^. Using different genetic approaches, we and another group have recently demonstrated that BASCs are authentic resident stem cells that differentiate, at population level, into multiple epithelial cell lineages^6,7^. In bronchiolar-injury model, BASCs give rise to bronchiole epithelial cells including club cells and ciliated cells for repair of distal airway (uni-directional: bronchiole). In alveolar-injury model, BASCs contribute to AT2 and AT1 cells to regenerate the alveoli (uni-directional: alveoli). However, whether single BASCs have the broader bi-directional differentiation potential to regenerate both distal airway and alveoli remains unknown. In this study, using in vivo single cell clonal analysis, we identified the bi-potency of single BASCs that differentiate into club cells, ciliated cells, AT2 and AT1 cells after bronchiole-alveoli double injuries.

To study the behavior of single BASCs after lung bronchiole-alveoli dual injury, we used a dual recombination derived multicolor fluorescence-reporter line named *R26-Confetti2*^6,8^. Different from conventional *R26-Confetti* reporter, a rox-Stop-rox cassette was inserted before confetti so that this allele could responds to two orthogonal recombinases, Dre and Cre. After dual Dre-rox and Cre-loxP recombination, Dre^+^Cre^+^ cells would express a single-color fluorescent protein out of three, RFP, YFP, GFP (Fig. 1a). We crossed *R26-Confetti2* with *Sftpc-DreER* and *Scgb1a1-CreER* and generated *Sftpc-DreER;Scgb1a1-CreER;R26-Confetti2* triple-positive mice and then treated them with a single low dose of tamoxifen for clonal analysis of single CC10^+^SPC^+^ double-positive BASCs. After 1 week of tamoxifen (Tam) injection at 7 weeks, lung samples were collected and sectional immunostaining of CC10 and SPC. Quantification of the fluorescence^+^ (RFP^+^ or YFP^+^ or nGFP^+^) BASCs showed that there are 148 fluorescence^+^ BADJ fields among 1189 BADJ fields, indicating that ~1 out of 8 BADJ fields, on average, harbors one fluorescent BASC. Therefore, the sparse labeling of BASCs by *R26-Confetti2* strengthens the clonality for single cell analysis of BASCs. Quantitatively, 93.92 % ± 0.79 % fluorescence^+^ BADJ fields contained only one single-color BASC (RFP^+^ or YFP^+^ or nGFP^+^). And 1.17% ± 0.59% fluorescence^+^ BADJ fields contained same-color BASCs (RFP^+^-RFP^+^ or YFP^+^-YFP^+^ or nGFP^+^-nGFP^+^) (Fig. 1b, c; Supplementary Fig. S1). Then we performed bronchiole-alveoli double injuries to investigate the roles of single BASCs in lung repair after injuries induced by naphthalene and bleomycin (Fig. 1d). In the control group treated with vehicle, fluorescent^+^ cells were still located at BADJs (Fig. 1e). In contrast, immunostaining on serial lung sections showed three distinct clone types: bronchiolar clones, alveolar clones, and bi-directional mixed clones containing both bronchiolar and alveolar epithelial cells (Fig. 1f, g; Supplementary Fig. S2, S3). The existence of mixed clone demonstrated the remarkable potential of a single BASC for bi-directional epithelial regeneration. However, the bi-directional potential cannot be detected in unilateral injury by either naphthalene or bleomycin treatment (Supplementary Fig. S4, S5).

**Fig. 1:**
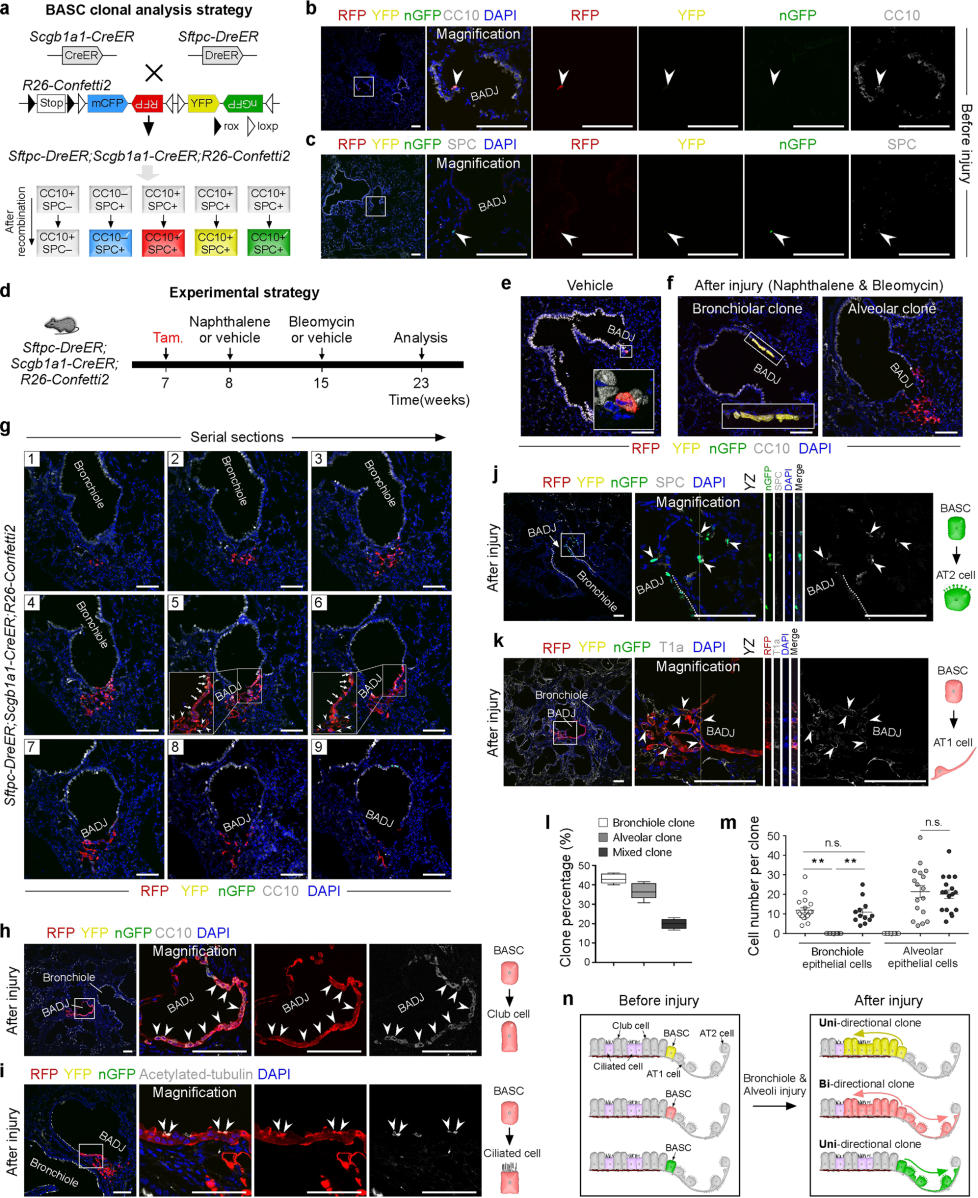
Identification of bi-directional potency of single BASCs for lung repair and regeneration. **a** Schematic figure showing strategy for labeling of single BASCs. **b**, **c** Lung sections stained for CC10 (b) or SPC (c). **d** Schematic figure showing the double injuries induced by naphthalene and bleomycin. Vehicle is used as control. **e** Immunostaining for CC10 on lung sections after vehicle treatment. **f** Immunostaining for CC10 on lung sections after double injuries shows that single BASCs differentiate into bronchiolar clone (left) or alveolar clone (right). **g** Serial sections (1–9) of a bi-directional mixed clone shows both bronchiolar and alveolar epithelial cells derived from single BASCs. **h**–**k** Immunostaining for CC10 (h), acetylated-tubulin (i), SPC (j) or T1a (k) on lung sections containing bi-directional mixed clones. **I** Quantification of the percentage of three distinct types of clones in injured lung. **m** Quantification the cell number of three types of clones. Data are mean ± s.e.m.; *P < 0.01, n.s. non-significant (left *P* = 0.1145; right *P* = 0.7161); Two-tailed *t* test. **n** Cartoon image shows that single BASCs expand to three types of clones after lung injury. Scale bars, 100 μm. Each image is representative of five individual samples

To further confirm the cell types of mixed clones, we performed co-immunostaining for fluorescent proteins and club cells marker CC10 or ciliated cells marker acetylated-tubulin. We found, in the bi-directional mixed clone, single BASC-derived cells contributed to both club cells and ciliated cells of bronchiole (Fig. 1h, i). Furthermore, co-immunostaining for fluorescent proteins and AT2 cells marker SPC or AT1 cells marker T1a showed that single BASCs also contribute to AT2 and AT1 cells in alveolar direction of the bi-directional mixed clones (Fig. 1j, k). We then quantified the three types of clones (uni- or bi-directional) in *Sftpc-DreER;Scgb1a1-CreER;R26-Confetti2* lungs after double-injuries and found that bronchiolar clones, alveolar clones and mixed clones constituted 43.22% ± 1.15%, 36.90% ± 1.89% and 19.86% ± 1.137% of all clones, respectively (Fig. 1l). The percentage of mixed clones was significantly higher than the labeling of fluorescence^+^ BADJ fields that harbor two BASCs with a common tag before injuries, indicating that most of the mixed clones after injury are derived from single BASCs with one tag. Quantification of the cell number of each clone showed that there was no significant difference between the contribution of single BASCs to bronchiolar epithelial cells in the uni-directional bronchiolar clones and that of the mixed clones (11.84 ± 1.34 in uni-directional bronchiolar clones versus 10.92 ± 1.79 in mixed clones; Fig. 1m). Further, there was also no significant difference in the contribution of single BASCs to alveolar epithelial cells in uni-directional alveolar clones compared with the mixed clones (21.41 ± 3.12 in uni-directional alveolar clones versus 20.01 ± 2.25 in mixed clones; Fig. 1m). Taken together, these data demonstrated that single BASCs have bi-directional differentiation potential to contribute to club cells, ciliated cells, AT2 and AT1 cells after lung injuries. Notably, the strength of bi-directional BASC in regenerating each directional epithelium was not compromised compared with other uni-directional clones.

Collectively, using in vivo single cell clonal analysis, this work provided genetic evidence that a single BASC has potential to differentiate into both bronchiolar and alveolar epithelial cells (bi-directional). Recently, using a split-intein effector recombination system, another study also specific labeled BASCs and reported a similar results that BASCs could contribute to new club and AT2 cells after both bronchiolar and alveolar damage induced by influenza virus infections^7^. However, in their study, BASCs are not labeled at single cell level for clonal analysis^7^. It remains unclear if one BASC or a group of BASCs at BADJ contribute to bi-directional epithelium. Using fluorescent reporter based on confetti strategy, we showed that single BASCs could contribute to four cell types, including club cells, ciliated cells, AT2 and AT1 cells after lung injuries. In the mixed clone of our injury strategy, the initial two daughter cells arising from a labeled single BASCs could have at least three models. The two daughter cells of the first model include a CC10^+^SPC^–^ club cell for responding to bronchiole injury and a maintaining CC10^+^SPC^+^ BASC for responding to next alveolar injury. The second model is that the single BASC divides into two BASCs, which could respond to airway and alveolar injuries, respectively. The third possible model is that the single labeled CC10^+^SPC^+^ BASC first divide into a CC10^+^SPC^–^ club cell and a CC10^–^SPC^+^AT2 cell for responding to the airway and next alveolar injuries respectively. The above models may happen many times during clone expansion and is hard to detect in vivo, which involves complex cell proliferation and differentiation. Nevertheless, all the progenies originated from the initial single BASC. Our recent work has showed that BASCs are heterogeneous and could be subdivided into two subpopulations, BASC-1 and BASC-2^6^. Whether bi- or uni-directional differentiation potential is pre-determined by different subpopulation is unclear. It is possible that both subpopulations of BASCs could be activated for bi-directional differentiation, or they could be inter-changeable to become more plasticity for bi-directional epithelium repair. Understanding the molecular mechanisms regulating BASCs proliferation and differentiation merits further investigation in future.

## Supplementary information

Supplementary information

## Supplementary information

**Supplementary Information** accompanies the paper at (10.1038/s41421-019-0132-8).
