# Supplementary material for: Bi-directional differentiation of single bronchioalveolar stem cells during lung repair
Source: Cell Discov. 2020 Jan 7;6:1. doi: 10.1038/s41421-019-0132-8 (PMC6952422; doi:10.1038/s41421-019-0132-8)

Supplementary Information

Bi-directional differentiation of single bronchioalveolar stem cells during lung repair

Kuo Liu, Muxue Tang, Qiaozhen Liu, Ximeng Han, Hengwei Jin, Huan Zhu, Yan Li, Lingjuan He, Hongbin Ji, Bin Zhou

Supplementary Methods

Supplementary Figure Legends

Supplementary Figure S1-S5.

Materials and Methods

Mice

All mouse experiments were strictly within the guidelines of the Institutional Animal Care and Use Committee (IACUC) of the Institute of Biochemistry and Cell Biology, Shanghai Institutes for Biological Sciences, Chinese Academy of Sciences. *Sftpc-DreER*, *Scgb1a1-CreER* and *R26-Confetti2* mouse lines were described previously[1-3]. All mice used of this study were kept at C57BL6 and C57BL6/ICR mixed backgrounds. Tamoxifen (Sigma, T5648) was dissolved in corn oil and stored in 4°C. Tamoxifen treatment to mice at the indicated time by oral gavage (0.025 mg/g).

Genomic PCR

The mouse tail was used to extract genomic DNA. Briefly, the tail tissues were lysed in the lysis buffer for more than 12 hours at 55°C and then the mixture was centrifuged at the speed of 20000 *rpm* for 6 min to obtain supernatant solution of the genomic DNA. Next, using isopropanol to precipitation of the DNA. And then using 70% ethanol to wash the DNA by centrifugation at 20000 *rpm* for 5 min. Finally, the DNA was dissolved in Dnase/Rnase free water. Genomic PCR was described previously[4].

Tissue collection and Immunofluorescent staining

Immunostaining protocol was according to previously described.[5] Briefly, after euthanasia of mouse, the lung tissues were collected in 6-well plates and then fixed in 4% paraformaldehyde (PFA, Sigma) for 60 min at 4°C. After washing several times using PBS, then the tissues were dehydrated at 30% sucrose (dissolved in PBS) overnight at 4°C. After the tissues sink to the bottom of well plates, then embedded in OCT (Sakura) and stored in -80°C. 12 μm frozen sections were collected on slides and then stored in -20°C. For immunofluorescent staining, tissue sections were first put in fume hood and dry by wind, and then remove OCT by washing in PBS. Next, the tissue sections blocked in 5% PBSST (0.1% Triton X-100 and 5% donkey serum in PBS) for 40 min at room temperature, then discard PBSST block buffer and incubated with primary antibodies at 4°C overnight in dark. Primary antibodies and its dilution were used: CC10 (Santa Cruz, SC-9772; 1:200), tdTomato (Rockland, 600-401-379; 1:1,000 dilution), acetylated tubulin (Sigma, T7451; 1:500 dilution), SPC (Millipore, AB3786; 1:200 dilution), T1a (DSHB, 8.1.1; 1:200 dilution). The next day, slides were washed in PBS for several times to remove primary antibodies and then incubated with secondary antibodies and DAPI (Vector Laboratories) for 40 min at room temperature in dark. Next, after washing in PBS for several times, the slides were mounted with mounting medium and stored in -20°C. The secondary antibodies were Alexa donkey anti-rabbit 555 (Invitrogen, A31572; 1:1000), Alexa donkey anti-mouse 647 (Invitrogen, A31571; 1:1000), Biotin-sp-a-rabbit IgG (JIR, 711-065-152; 1:200), Biotin-sp-a-goat IgG (JIR, 705-065-147; 1:200), Biotin-sp-goat anti-Syrian Hamster IgG(H+L) (JIR, 107-065-142; 1:200), Dylight 647-streptavidin (JIR, 016-490-084; 1:200). Immunostaining images were acquired by Olympus confocal microscopy system (FV1200). The ImageJ (NIH) software was used to analyze images.

Bronchiole-Alveoli double injury

Bronchiolar injury was achieved by naphthalene intraperitoneal injection and alveolar injury was induced by intratracheal instillation of bleomycin, as previously described[1]. Naphthalene (Sigma 84679) was dissolved in sterile corn oil and the final concentration was 25 mg ml^{-1}. Bleomycin (Sigma B8416) was freshly dissolved in PBS (Invitrogen, 10010049) and the final concentration was 10 U ml^{-1}. The 10 U ml^{-1} bleomycin was stored at –80°C and diluted to 1 U $ml^{-1}$with PBS before use. For bronchiole-Alveoli double injuries, *Sftpc-DreER;Scgb1a1-CreER;R26-Confetti2* mice were treated with tamoxifen at 7 weeks, and then treated with 250 mg kg^{-1} naphthalene or vehicle (corn oil) at 8 weeks for bronchiolar injury. Then after bronchiolar recovery for 7 weeks, the mice were treated with 2 U kg^{-1} bleomycin or vehicle (PBS) for alveolar injury and then the lung were collected after another 8 weeks. For clonal analysis of single BASCs after bronchiole-Alveoli double injuries, single string of $fluorescence^{+}CC10^{+}$ club cell number or $fluorescence^{+}$acetylated-$tubulin^{+}$ciliated cell number was quantified for each BADJ field. And single $fluorescence^{+}SPC^{+}$ AT2 cell or single $fluorescence^{+}T1a^{+}$ AT1 cell number was quantified for each BADJ field.

Statistics analysis

All data were acquired in this study from four to five independent experiments as indicated in figure legends and presented as mean values ± s.e.m. Two-sided unpaired Student's t-test was used for statistical comparison between two groups. $P < 0.05$ was accepted for statistically significant.

References

1 Liu Q, Liu K, Cui G *et al.* Lung regeneration by multipotent stem cells residing at the bronchioalveolar-duct junction. *Nature Genetics* 2019.

2 Han X, Wang Y, Pu W *et al.* Lineage Tracing Reveals the Bipotency of SOX9+ Hepatocytes during Liver Regeneration. *Stem Cell Reports* 2019; **12**:624-638.

3 Rawlins EL, Okubo T, Xue Y *et al.* The role of Scgb1a1+ Clara cells in the long-term maintenance and repair of lung airway, but not alveolar, epithelium. *Cell Stem Cell* 2009; **4**:525-534.

4 Liu K, Yu W, Tang M *et al.* A dual genetic tracing system identifies diverse and dynamic origins of cardiac valve mesenchyme. *Development* 2018; **145**.

5 Zhang H, Huang X, Liu K *et al.* Fibroblasts in an endocardial fibroelastosis disease model mainly originate from mesenchymal derivatives of epicardium. *Cell Res* 2017; **27**:1157-1177.

Supplementary Figure legends

Supplementary, Fig S1. Specific labeling of single BASCs by *Sftpc-DreER;Scgb1a1-CreER;R26-Confetti2* triple-positive line. **a** Schematic showing experimental strategy. **b** Quantification of the percentage of BADJ fields which containing single-color (RFP^+ or YFP^+ or $nGFP^+$) BASC or same-color BASCs (RFP^+-RFP^+ or YFP^+-YFP^+ or $nGFP^+$-$nGFP^+$) over total $fluorescent^+$ BADJ fields. **c** Quantification of the percentage of each single-color BASC (RFP^+ or YFP^+ or $nGFP^+$) detected in single-color BADJ field. Data are mean ± s.e.m. Two-tailed *t* test. N = 3 biologically independent mice.

Supplementary, Fig S2. Bronchiolar clone from single BASCs after double injuries. **a** Schematic showing strategy for naphthalene (or vehicle) and bleomycin (or vehicle) injuries. **b** Immunostaining for CC10 on serial lung sections shows that single BASCs differentiate into bronchiole epithelial cells. Scale bars, 100 μm. Each image is representative of five individual samples.

Supplementary, Fig S3. Alveolar clone from single BASCs after double injuries. **a** Schematic figure showing strategy for naphthalene (or vehicle) and bleomycin (or vehicle) injuries. **b** Staining on serial lung sections shows that single BASCs differentiate into alveolar epithelial cells. Scale bars, 100 μm. Each image is representative of five individual samples.

Supplementary, Fig S4. Single BASCs contribute to bronchiolar epithelial cells but not alveolar epithelium after naphthalene-induced bronchiolar injury. **a** Schematic figure showing the naphthalene injury strategy. **b** Co-Immunostaining of fluorescence proteins and CC10 on lung sections after naphthalene treatment shows that single BASCs contribute to club cells. **c** Co-Immunostaining of fluorescence proteins and acetylated-tubulin show that single BASCs contribute to ciliated cells. Scale bars, 100 μm. Each image is representative of three individual samples.

Supplementary information, Fig S5. Single BASCs contribute to alveolar epithelial cells but not bronchiolar epithelium after bleomycin-induced alveolar injury. **a** Schematic figure showing the bleomycin injury strategy. **b** Co-Immunostaining of fluorescence proteins and SPC on lung sections after bleomycin treatment shows that single BASCs contribute to AT2 cells. **c** Co-Immunostaining of fluorescence proteins and T1a shows that single BASCs contribute to AT1 cells. Scale bars, 100 μm. Each image is representative of three individual samples.

Supplementary, Fig. S1

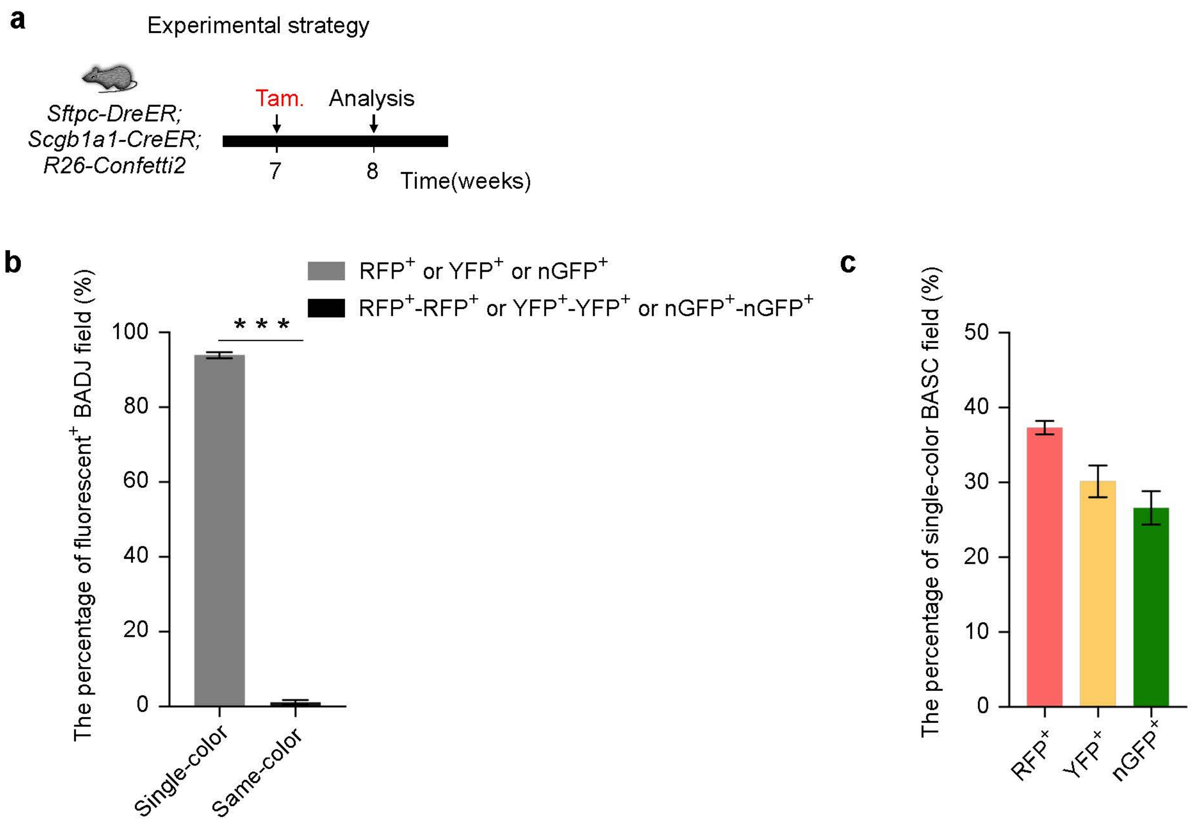

Supplementary, Fig. S2

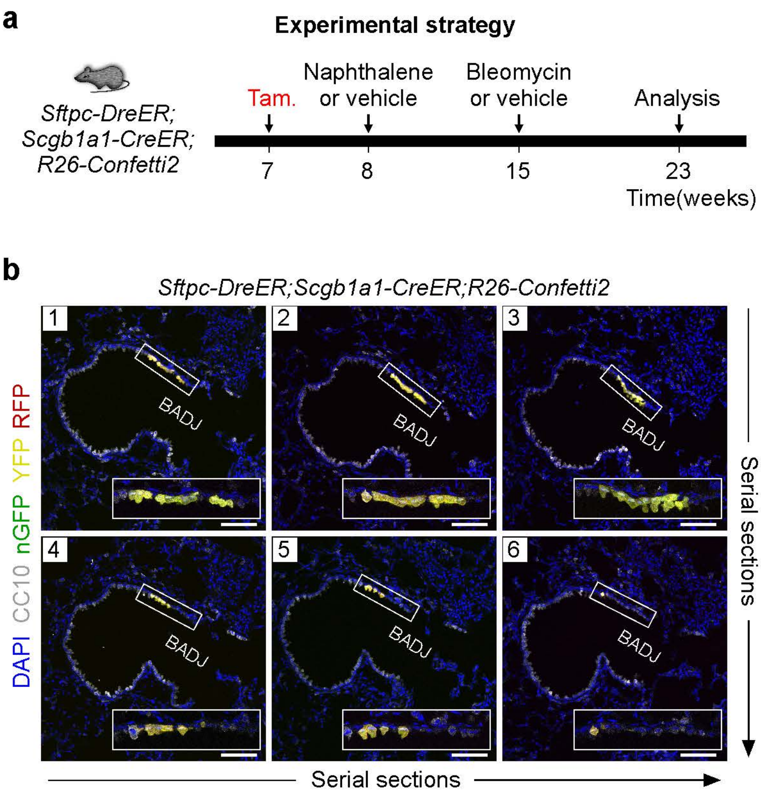

Supplementary, Fig. S3

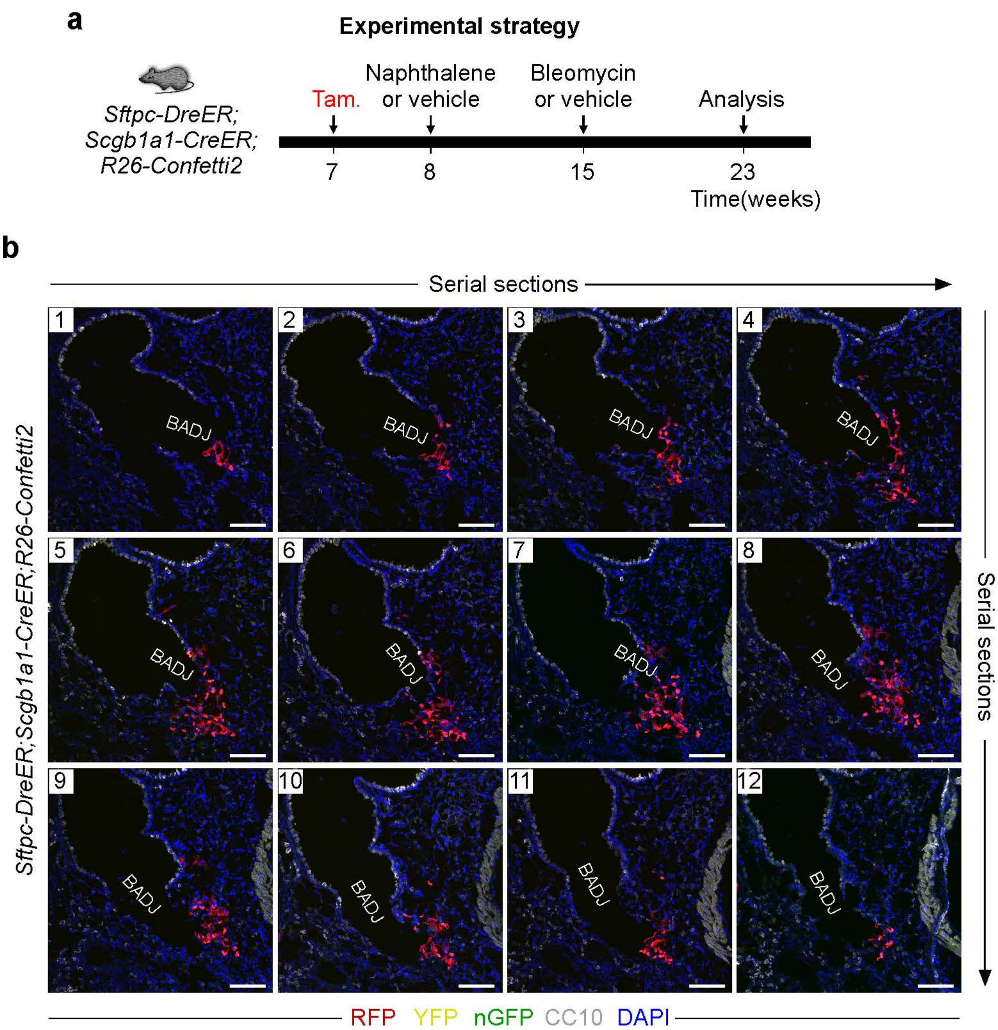

Supplementary, Fig. S4

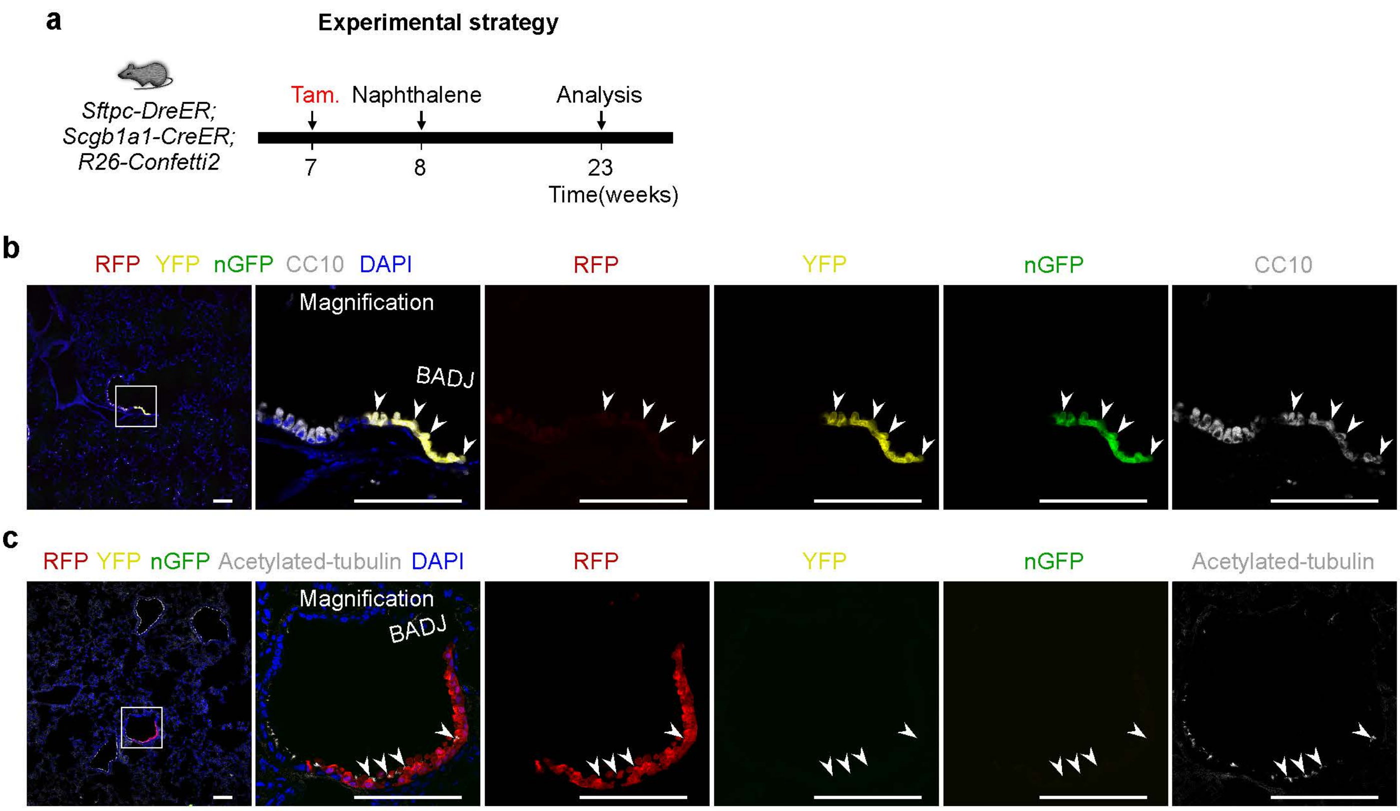

Supplementary, Fig. S5

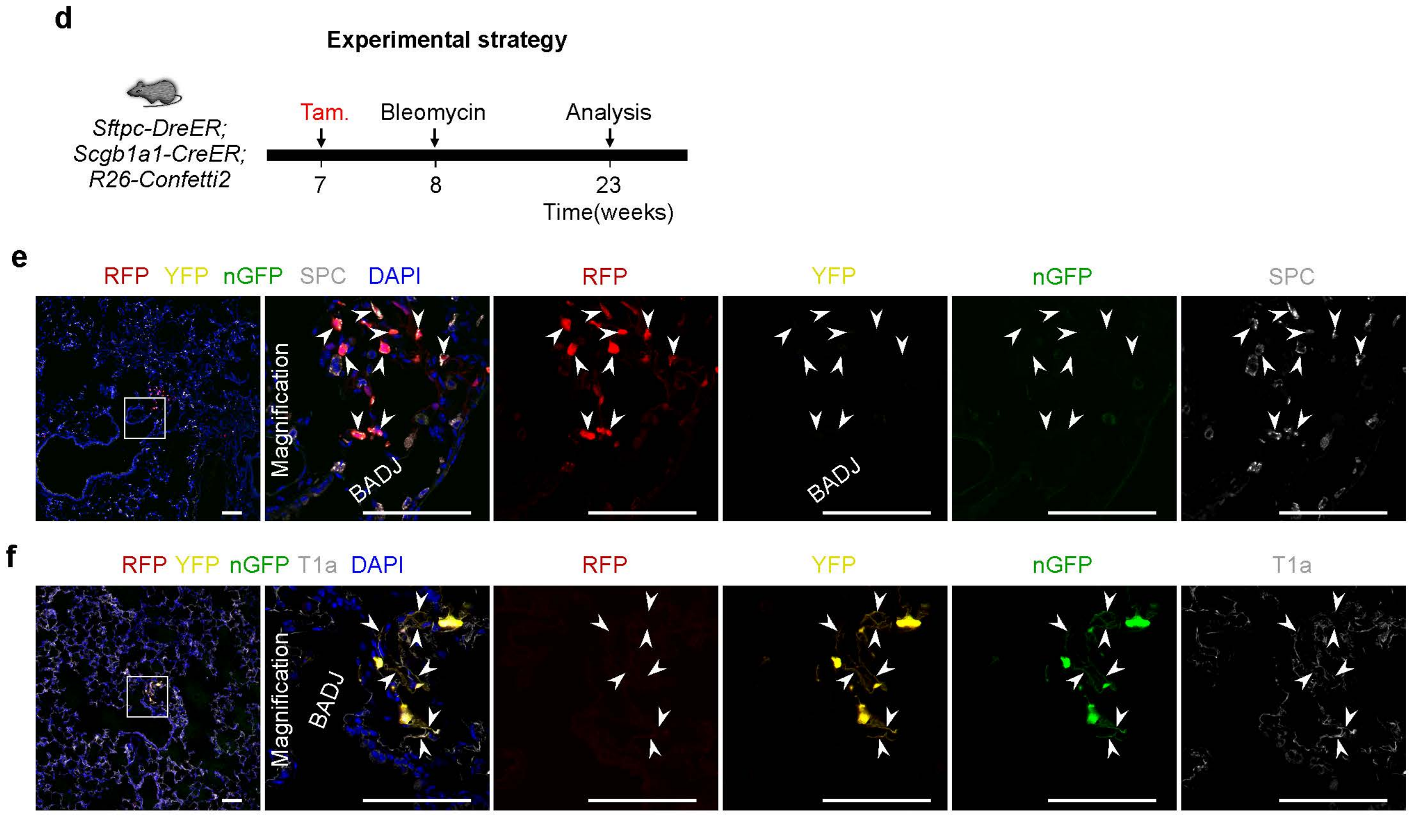

Supplement: Supplementary file 1 — Supplementary information [file 41421_2019_132_MOESM1_ESM.pdf]
